# Umami and saltiness enhancements of vegetable soup by enzyme-produced glutamic acid and branched-chain amino acids

**DOI:** 10.3389/fnut.2024.1436113

**Published:** 2024-08-19

**Authors:** Kiyota Sakai, Masamichi Okada, Shotaro Yamaguchi

**Affiliations:** Innovation Center, Amano Enzyme Inc., Kakamigahara, Japan

**Keywords:** protease, glutaminase, saltiness enhancer, glutamic acid, branched-chain amino acid

## Abstract

**Introduction:**

One major challenge of reducing salt content in food is the risk of the overall taste becoming bland. Enhancing saltiness is an effective strategy for salt reduction, and the development of salt-reduced foods using these saltiness-enhancing flavorants as food additives is underway. However, an increasing number of consumers demand a reduction in additives in clean-label foods.

**Objective:**

Enzyme processing of food is an attractive strategy for developing clean-label foods because enzymes are not considered additives. We aimed to improve the saltiness and umami intensity of vegetable soups by enzyme treatment while meeting clean-label requirements. We first optimized the enzymatic reaction conditions of a protease and glutaminase blend and then investigated the synergistic effects of this enzyme blend on the taste of vegetable soup.

**Results:**

Sensory evaluations indicated that the reaction products (e.g., protein hydrolysates or amino acids) could enhance the umami, kokumi, and saltiness intensity of vegetable soup supplemented with 0.5% NaCl. Notably, the saltiness intensity ratio of the enzyme-treated soup with 0.50, 0.45, and 0.40% NaCl were increased by 1.31-, 1.16-, and 0.99-fold, respectively, when this ratio for the control soup with 0.50% NaCl was set to 1.0. This indicates a 20% salt reduction rate can be achieved by enzyme treatment. Moreover, we found that these enhancements were synergically caused by enzyme-produced glutamic acid and branched-chain amino acids.

**Conclusion:**

Our findings suggest that using enzyme blends of bacterial and fungal proteases and glutaminase is an effective approach to enhancing the saltiness levels of vegetable soups while meeting clean-label requirements.

## 1 Introduction

Saltiness is the taste conferred by sodium and other ions in food. It not only contributes to delectability but is also routinely used to emphasize the taste of food (1). However, higher sodium intake is often associated with an increased risk of high blood pressure (2, 3). People in developed countries consume 9–12 g of NaCl per day, which far exceeds the recommended amount of 5 g/day (4, 5). Thus, reducing salt intake is essential for preventing the onset and aggravation of hypertension and improving longevity (6–9). However, one of the major challenges of salt reduction is that the taste of the food can become bland.

The saltiness enhancement effect (taste–taste and odor–taste interactions) by adding certain flavorants (tastants and odorants) is an effective strategy for salt reduction. Various saltiness-enhancing tastants have been reported: amino acids (e.g., acidic amino acids, basic amino acids, and branched-chain amino acids), peptides (e.g., arginylproline), nucleotides (e.g., guanosine monophosphate and inosine monophosphate), sour organic acids (e.g., tartaric acid), and some spicy substances (e.g., spilanthol) (10–15). Also, several saltiness-enhancing odorants have been reported: pyrazine compounds (e.g., 2-ethyl-3,5-dimethyl pyrazine), fatty alcohols (e.g., 1-octen-3-ol), fatty aldehydes (e.g., 3-(methylthio) propanal), and sulfur-containing compounds (e.g., 1-(2-furyl)ethanethiol, 2-furfuryl mercaptan) (16–19). The development of salt-reduced foods using these saltiness-enhancing flavorants as food additives is currently in progress. In particular, glutamic acid and yeast extract have been extensively studied and commercialized as food additives that enhance saltiness in various foods (20). Although this is an effective strategy, the increasing consumer demand for clean-label food necessitates the reduction of additives (21). To meet these demands, the scientific community and food industry are attempting to develop more acceptable strategies for reducing salt content while maintaining saltiness levels.

Various industrial enzymes have been used in food processing and ingredient production to improve the physical, nutritional, and/or sensory properties of food (22–25). Recently, enzyme catalysis in food processing has received significant attention as an effective strategy for producing clean-label foods because enzymes in a denatured or inactivated form in the final product are not considered as additives (21, 25–28). Therefore, taste modification using enzymes is an attractive approach for increasing the umami or saltiness levels of clean-label foods. Various previous studies reported amino acids and peptides with umami and enzymes synthesizing them: glutamic acid synthesized by glutaminase (EC 3.5.1.2), γ-glutamyl peptides synthesized by γ-glutamyltranspeptidase (EC 2.3.2.2), lactoyl-peptides synthesized by lactase (EC 3.2.1.108) or other endogenous enzymes derived from microorganisms, and succinyl amino acids synthesized by transsuccinylase (EC 2.3.1.109) (29). Whereas, relatively few previous studies reported saltiness or saltiness-enhancing amino acids and peptides and enzymes synthesizing them: protein hydrolysates synthesized by proteases (EC 3.4.-.-), peptide-based Maillard reaction products by proteases and heat treatment, γ-glutamyl peptides synthesized by γ-glutamyltranspeptidase (30–32). However, the effects of these enzymes and their products in possibly enhancing the umami or saltiness of actual foods are largely unclear.

The present study aimed to develop modification methods using food-grade enzymes to improve the saltiness levels of vegetable soup while meeting clean-label requirements. Three types of enzymes were used in this study: bacterial and fungal proteases and glutaminase. Extracellular proteases catalyze the breakdown of proteins into smaller polypeptides or single amino acids (33). Bacterial proteases mainly contain *endo*-peptidases, which degrade proteins to larger peptides, while fungal proteases mainly contain *exo*-peptidases, which degrade larger peptides to smaller peptides and amino acids (33). One possible adverse effect using proteases in food industry is the bitterness development especially plant-derived peptides might display bitterness owing to the higher proportion of hydrophobic amino acids in plant proteins (34–36). In addition, glutaminase is an enzyme that catalyzes the deamidation of glutamine (Gln) to glutamic acid (Glu) and is widely used for improving the umami taste of foods (37). In general, umami enhancement by Glu molecule can mask the intensity of bitterness in foods (38). We optimized the ratio of these three enzymes and their reactions to produce glutamic acid and then investigated the synergistic effects and salt reduction rate of the enzyme blend in enhancing the umami and saltiness levels of vegetable soup. Subsequently, we analyzed the enzymatically generated amino acids and evaluated their saltiness-enhancing effects by taste–taste interactions. Finally, we investigated the ability of the reaction products by proteases and glutaminase to mask the certain bitterness of potassium chloride (KCl) as a salt replacer.

## 2 Materials and methods

### 2.1 Materials

Concentrated vegetable soup (Kagome Co., Ltd., Tokyo, Japan) and salt were obtained from a local supermarket in Nagoya (Japan). The nutritional content of the soup was 161 kcal, 1.9% protein, 0.4% lipid, and 37.5% carbohydrate. Soy protein isolate was purchased from Fuji Oil Co., Ltd. (Osaka, Japan). Bacterial and fungal proteases and bacterial glutaminase (Amano Enzyme Inc., Nagoya, Japan) were commercially available food-grade products (Thermoase PC10FA, Protease “Amano” A, and Glutaminase SD-C100S).

### 2.2 Preparation of vegetable soup

In this study, we selected commercially available concentrated vegetable soups to minimize the experimental errors caused by the variety, quality, harvest time, and storage conditions of vegetables and the cooking methods of the soup. This soup is made from a mixture of onions, carrots, celery, and broccoli. According to the manufacturer’s recipe, an appropriate concentration of vegetable soup was prepared by diluting the concentrated soup with water (1:20 mass-to-volume ratio). We optimized three enzyme ratios to maximize the produced Glu amounts by enzymes (Supplementary Figure S1). Enzyme assays were performed in the mixtures containing 50 mM phosphate buffer (pH 6.0), 5% soy protein isolate, and various concentrations of each enzyme. Enzymatic reactions were incubated at 50°C for 1 h. As a preliminary result, combination ratios of 30–40 U/g-protein bacterial protease and 16,000–20,000 U/g-protein fungal protease maximized the total amounts of Gln + Glu at approximately 2 mg/g-protein (Supplementary Figures S1A,B). Then, as a result of adding various concentrations of glutaminases to the two optimized protease blends, 0.03–0.09 U/g-protein glutaminase maximized the amount of produced Glu amounts (Supplementary Figure S1C). Based on these preliminary results, the prepared soup was treated with bacterial and fungal proteases (30 and 1,600 U/g-protein, respectively) and glutaminase (0.03 U/g-protein) at 50°C for 1 h with agitation. The enzyme activities were referred to as product information. When evaluating the effect of the enzyme blends on the taste of soup containing NaCl or KCl, at the same time as the enzyme addition, NaCl was added to the soup at the final concentrations of 0.35–0.50%, or KCl was added to the soup at the final concentrations of 0.30% or 0.50%. After its reaction, the enzyme-treated soup was heated at 90°C for 10 min to deactivate the enzymes. It was cooled to 50°C before being used for further analyses. Moreover, when investigating the effects of additional amino acids on saltiness enhancement through taste–taste interactions, these amino acids were added to the soup at the following concentrations: 20.9 μmol/L His, 122.8 μmol/L Asp, 205.8 μmol/L Glu, 146.7 μmol/L Ser, 225.6 μmol/L Ala, 75.4 μmol/L Gly, 93.3 μmol/L Val, 76.1 μmol/L Leu, and/or 68.7 μmol/L Ile. The soup was incubated at 50°C for 1 h with agitation and was finally heated at 90°C for 10 min.

### 2.3 Residual activity of enzymes after deactivation process

The residual activity of protease was assayed in 6 mL mixtures containing 50 mM phosphate buffer (pH 6.0), 5% soy protein isolate, and enzyme-contained soup after the deactivation process. This reaction solution was incubated at 50°C 4 h and added to 5 mL 0.44 mol/L trichloroacetic acid solution. After the incubation at 37°C for 30 min, the TCA-treated solution was centrifuged at 15,000 × g for 10 min. Then, 5 mL 0.55 mol/L sodium carbonate solution and 1 mL Folin & Ciocalteu’s Phenol Reagent (Fujifilm Wako Pure Chemical Corporation, Osaka, Japan) were added to 2 mL supernatant and incubated at 37°C for 30 min. The absorbance of the reaction solution was measured at 660 nm. One unit of protease activity was defined as the amount of enzyme required to produce 1 μg of amino acids per minute.

The residual activity of glutaminase was assayed in 2 mL mixtures containing 50 mM phosphate buffer (pH 6.0), 2% Gln, and enzyme-contained soup after the deactivation process. This reaction solution was incubated at 37°C 4 h. Produced Glu amounts were measured by L-Glutamate KIT YAMASA NEO according to the manufacturer’s instructions (Yamasa Corporation, Chiba, Japan). One unit of glutaminase activity was defined as the amount of enzyme required to produce 1 μmol of Glu molecule per minute.

### 2.4 Amino acid analysis

The amino acids produced by the enzymes were quantified using an Agilent 1,100 HPLC system (Agilent Technologies, Santa Clara, CA, United States) equipped with a fluorescence detector (excitation 230 nm and emission 450 nm PMT 9 or excitation 266 nm and emission 305 nm PMT 7). The supernatant was treated with *o*-phthalaldehyde and 9-fluorenylmethylchloroformate and separated on a Zorbax Eclipse-AAA column (4.6 × 150 mm, Agilent) with a gradient of 20 mM Na_2_HPO_4_ buffer (pH 8.2) and a 45:45:10 methanol:acetonitrile:H_2_O mixture for 20 min at a flow rate of 0.65 mL/min (39).

### 2.5 Peptide analysis

To precipitate protein molecules, 990 μL enzyme-treated and control vegetable soups were treated with 10 μL 10% trichloroacetic acid solution and these soups with a final concentration of 0.1% TCA were centrifuged at 15,000 × g for 10 min. The enzyme-produced peptides in the supernatants were analyzed using HPLC (Nexera X2, Shimadzu, Kyoto, Japan) equipped with a UV detector (220 nm). Each injection volume was 10 μL. The peptides were separated using the TSKgel G2000SWXL (300 × 7.8 mm I.D., Tosoh Corporation, Tokyo, Japan) with isocratic elution of 45% acetonitrile containing 0.1% trifluoroacetic acid for 25 min at a flow rate of 0.7 mL/min and 40°C.

### 2.6 Free amino nitrogen analysis

Enzyme-treated and control vegetable soups were centrifuged at 15,000 × *g* for 10 min, and their supernatants were deproteinized with a Nanosep^®^ Centrifugal Device (Pall Corporation, Port Washington, NY, United States) with a molecular weight cut-off of 30,000 Da. The amount of free amino nitrogen (FAN) was measured using the ninhydrin method (40).

### 2.7 Sodium ion analysis

Sodium ions were quantified using a LAQUAtwin-Na-11 pocket meter (Horiba Ltd., Kyoto, Japan). To quantify sodium concentration, standard curves were generated using 0.01, 0.1, 1, 2.0, and 2.5% NaCl solution at 25°C. Subsequently, the sodium ion concentration in the vegetable soup was spiked and analyzed under the same conditions.

### 2.8 Color analysis

The color of the vegetable soup was determined using a colorimeter (Chroma Meter CM-700d/600d; Konica Minolta, Tokyo, Japan) (41). The color analysis results were expressed according to the Commission International de l’Eclairage (CIE) system and reported as Hunter L* (lightness), a* (redness), and b* (yellowness).

### 2.9 Sensory tests

The sensory taste of the vegetable soup was evaluated by 12 employees (seven men and five women aged 25–55 years) of Amano Enzyme Inc., trained in sensory evaluation. These sensory panelists were selected based on their ability to perceive five basic tastes (umami, saltiness, sweetness, bitterness, and sourness) according to ISO 8586:2023 (42). The terms of five basic tastes were defined in this standard. Whereas the term kokumi was defined as a perceived thickness, mouthfulness, continuity, and depth, with reference to the previous report (43). These vocabularies are defined in ISO 8586:2023 or ISO 5492:2008 (44). For the tastes of vegetable soup, the samples were evaluated for four attributes (umami, saltiness, bitterness, and kokumi). The samples were scored on a structured scale from −2 (weak) to +2 (strong) relative to the control soup (non-treated soup), which was scored as 0. For the smell of vegetable soup, the samples were evaluated for only one attribute (smell). The samples were similarly scored on a structured scale from −2 (unpleasant odor) to +2 (pleasant fragrance). The Umami and saltiness ratio of the enzyme-treated vegetable soup was scored on an unstructured scale relative to the control soup (non-treated soup), in which the ratio was 1. To evaluate this test more accurately, the sensory panelists were trained using NaCl solution in 0.05% increments.

### 2.10 Statistical analysis

Vegetable soup in five different lots was used in this study. Data are presented as the mean ± standard error of five independent experiments to evaluate the effects of different sample formulations and preparations. Statistical differences among multiple groups were determined by one-way analysis of variance (ANOVA), followed by Tukey’s multiple comparison test. Statistical differences between the curves were analyzed using a two-way analysis of variance (ANOVA) followed by Tukey’s multiple comparison test. Tukey’s test at significance levels of 95% (*p* < 0.05) and 99% (*p* < 0.01) was used to determine the significant differences in the results. Heatmap and clustering analysis were performed using Heatplus in the Bioconductor package (version 3.19).

## 3 Results and discussion

### 3.1 Characteristics of enzyme-treated vegetable soup

**Figure 1:**
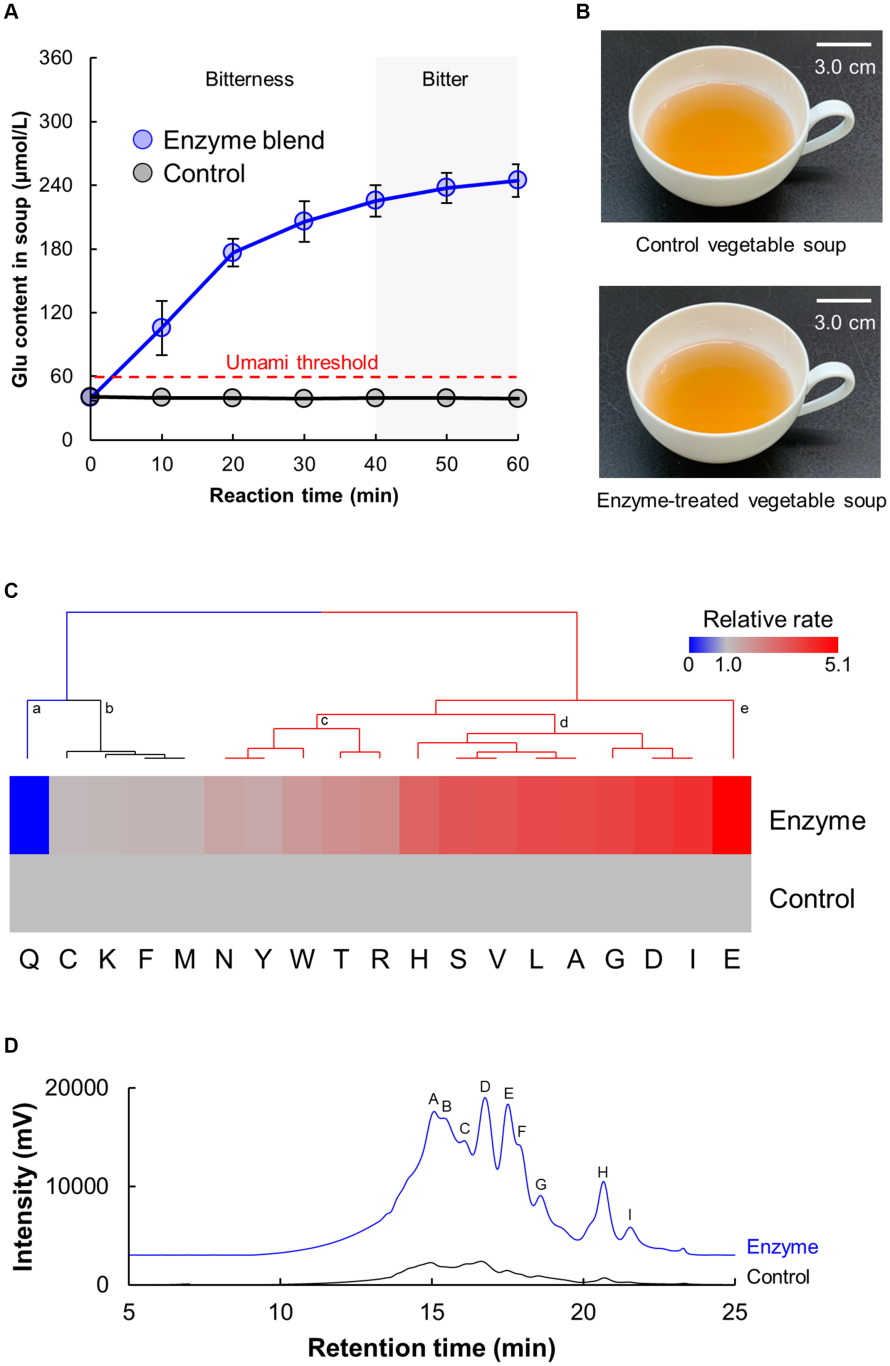
Vegetable soup produced by a blend of proteases and glutaminase. **(A)** Time-course of the Glu content produced by the enzyme blend. **(B)** Appearance of control and enzyme-treated vegetable soup. **(C,D)** Composition analysis of protein hydrolysates produced by the enzyme blend. Free amino acid **(C)** and peptide **(D)** profiles analyzed using HPLC. **(C)** Heatmap and cluster analysis of free amino acid profile in vegetable soup. **(D)** Size exclusion chromatography of free peptides in vegetable soup.

To maximize the umami intensity of the vegetable soup, glutamic acid (Glu) content was determined as an indicator (Figure 1A). The vegetable soup was treated with an optimized blend of protease and glutaminase for 60 min. The Glu content in the control soup remained constant at 40.0 μmol/L, which was below the panelists’ threshold Glu concentration (90 μmol/L) that our panelists required for sensing umami taste. In general, the Glu threshold for humans to perceive umami is 63 μmol/L (45). In contrast, Glu content in the enzyme-treated soup increased in a time-dependent manner and reached a plateau at 250 μmol/L. However, soups tended to become bitter when the reaction time exceeded 30 min in simple sensory tests. Generally, owing to the higher proportion of hydrophobic amino acids in plant-derived proteins, the peptides produced by enzymatic hydrolysis can elicit bitterness (34–36). Therefore, in the subsequent experiments, the reaction time for the enzyme blend was set to 30 min. The activities of proteases and glutaminase in the vegetable soup were not detected after the deactivation process. Furthermore, the reactivity of the enzyme blend did not differ among the five lots of vegetable soup.

Figure 1B shows the appearance of control and enzyme-treated soups. Other possible adverse effect of processing vegetable soup using proteases is color change via the Maillard reaction caused by free amino acids. However, both soups were visually similar in color. To investigate the color properties in detail, the objective color attributes of the proteins were characterized using L*a*b* coordinates. The L* (whiteness), a* (redness), and b* (yellowness) values of the control soup were 48.10 ± 0.19, 3.93 ± 0.01, and 18.76 ± 0.10, whereas those of the enzyme-treated soup were 48.22 ± 0.18, 3.93 ± 0.01, and 18.83 ± 0.15. This finding indicated that both soups have similar color properties.

### 3.2 Protein hydrolysates in enzyme-treated vegetable soup

To investigate the protein hydrolysates produced by the blend of proteases and glutaminase, free amino acids and peptides in the vegetable soup were measured using HPLC analysis (Figures 1C,D). Figure 1C shows the heatmap and cluster analysis of the increased rate of enzyme-treated soup compared with the amino acid content in the control soup. The relative free amino acid compositions were clustered into five different groups according to Euclidean distance: (a) significantly lower cluster, (b) no change cluster, (c) slightly higher cluster, (d) significantly higher cluster, and (e) significantly highest cluster. As evident from the clustering, most amino acids were enhanced. Total amino acid amounts of control and enzyme-treated soup were 730.4 ± 24.2 and 1252.8 ± 87.3 μmol/L, respectively. Interestingly, amino acids classified into cluster (d) showed that the amounts of branched-chain amino acids (BCAAs) such as Ile, Leu, and Val and other umami acidic amino acids such as Asp were increased by enzymatic reactions. Moreover, the Glu content in vegetable soup significantly increased with a decrease in Gln content. Figure 1D shows the results of size exclusion chromatography for separating peptides from vegetable soup by molecular weight. As evident from the chromatography results, enzyme treatment substantially enhanced the amount of total peptides in the vegetable soup. The same trend was observed for the FAN amounts, which control and enzyme-treated soup had 11.0 ± 0.3 and 39.9 ± 2.0 mg/L, respectively. Nine peaks were detected in vegetable soup hydrolyzed by protease, and the estimated molecular weights from peaks A to H were 7.6, 7.1, 6.0, 5.6, 4.8, 4.4, 3.6, 1.2, and 0.6 kDa, respectively. The estimated molecular weight of peak H was equal to at least the size of a tri-to-pentapeptide. These findings indicate that proteases substantially produce specific amino acids and lower molecular weight peptides from proteins in vegetable soup, and glutaminase converts free Gln into Glu with an umami taste.

The type and amount of free amino acids are important factors affecting human nutrition and food palatability (45–47). Regarding nutrition, five of the nine essential amino acids (Trp, Thr, Ile, Leu, and Val) were increased by enzyme treatment (Figure 1C). BCAAs are proteinogenic amino acids that account for 35% of the essential amino acids in muscle proteins and 40% of preformed amino acids required by mammals (48). Because not all ingested food proteins can be degraded into amino acids (49, 50), increasing the content of essential amino acids in food by enzyme treatment can potentially boost the amount of amino acids metabolized as nutrients. Regarding taste, of the 14 amino acids increased by the enzyme blend, nine (Asn, Tyr, Trp, Thr, Arg, His, Val, Leu, and Ile) have bitter taste, three (Ser, Ala, and Gly) have sweet taste, and two (Asp and Glu) have umami taste (45). However, the concentrations of all amino acids except Glu were lower than the threshold for sensing bitter, sweet, and umami tastes. Therefore, only Glu contributed to the umami taste of the soup.

### 3.3 Sensory evaluation of enzyme-treated vegetable soup with added sodium chloride

**Figure 2:**
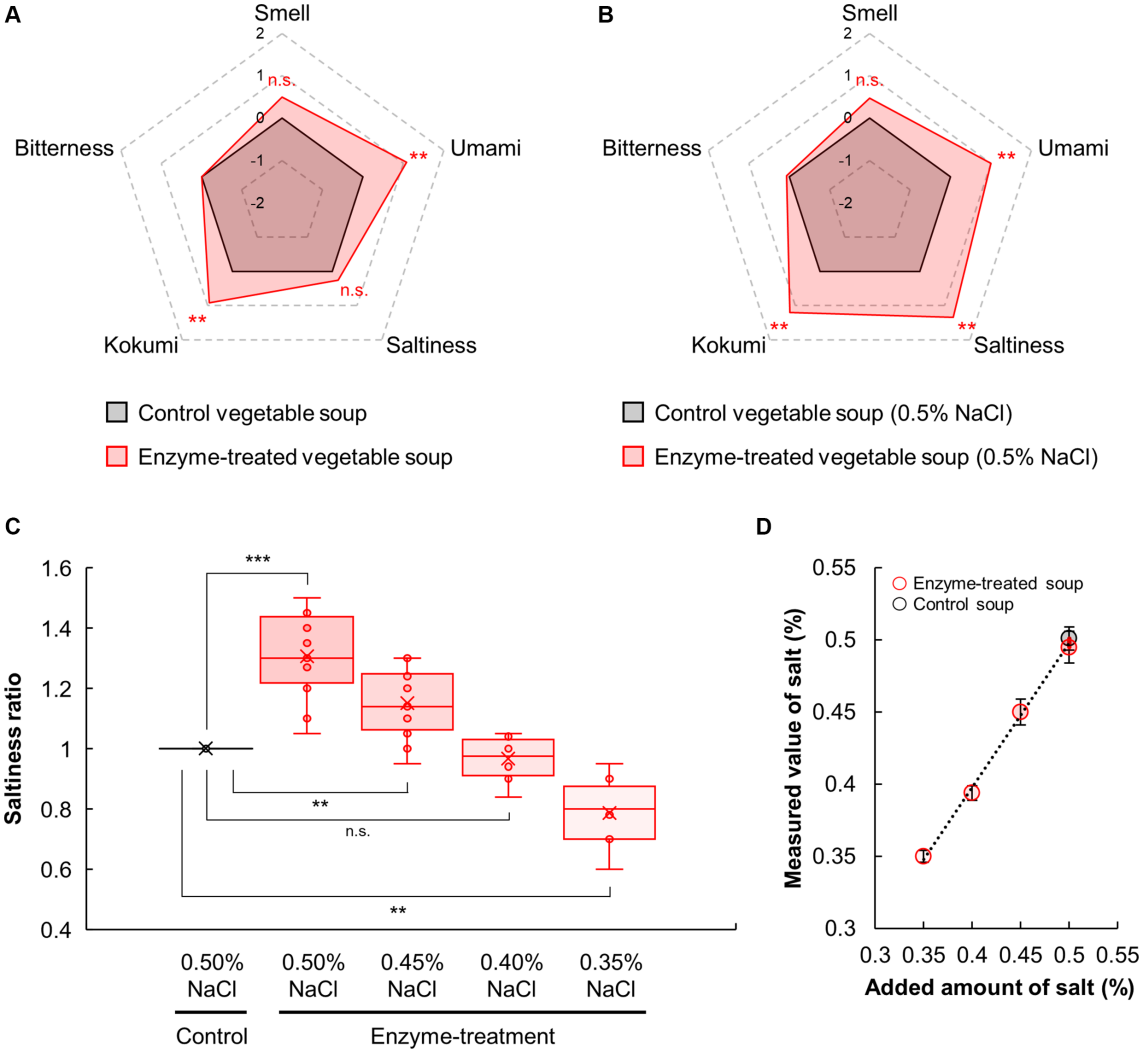
Sensory profiles of vegetable soup after NaCl addition. Sensory evaluations of control and enzyme-treated vegetable soups conducted in the absence **(A)** and presence **(B)** of 0.5% NaCl final concentration. **(C)** Salt reduction rate of vegetable soup. Sensory evaluations of enzyme-treated vegetable soup in the presence of 0.35, 0.40, 0.45%, and −0.50% NaCl compared with the saltiness of the control soup. **(D)** NaCl contents in the vegetable soup.

The effects on the sensory properties of vegetable soup were assessed using sensory evaluation. As shown in Figure 2A, the umami and kokumi levels of the enzyme-treated vegetable soup were significantly higher (*p* < 0.01) and the saltiness and smell of the enzyme-treated soup were slightly higher (*p* > 0.01) than those of the control soup. Here, regardless of enzyme addition, NaCl contents remained unchanged, which control and enzyme-treated soup had 0.33 ± 0.02 and 0.34 ± 0.04%, respectively. Although one possible adverse effect of using protease is bitterness development, the bitterness score of both vegetable soups was the same. Thus, the proteases and glutaminase blend could enhance the umami and kokumi tastes of the vegetable soup without causing bitterness.

Next, because the saltiness score of the enzyme-treated soup was slightly higher than that of the control soup, the ability of enzyme treatment to enhance the saltiness intensity of NaCl was evaluated. As shown in Figure 2B, 0.5% NaCl supplementation significantly increased the umami and kokumi levels in the enzyme-treated vegetable soup compared with the control vegetable soup (*p* < 0.01); however, bitterness was not significantly different between the two groups (*p* > 0.05). This was consistent with the results obtained for vegetable soup without NaCl addition (Figure 2A). Interestingly, in the presence of 0.5% NaCl, the saltiness of the enzyme-treated vegetable soup was significantly higher (*p* < 0.01) than that of the control soup. Thus, reaction products such as protein hydrolysates or amino acids produced by a protease and glutaminase blend can enhance the saltiness of NaCl in vegetable soups.

**Figure 3:**
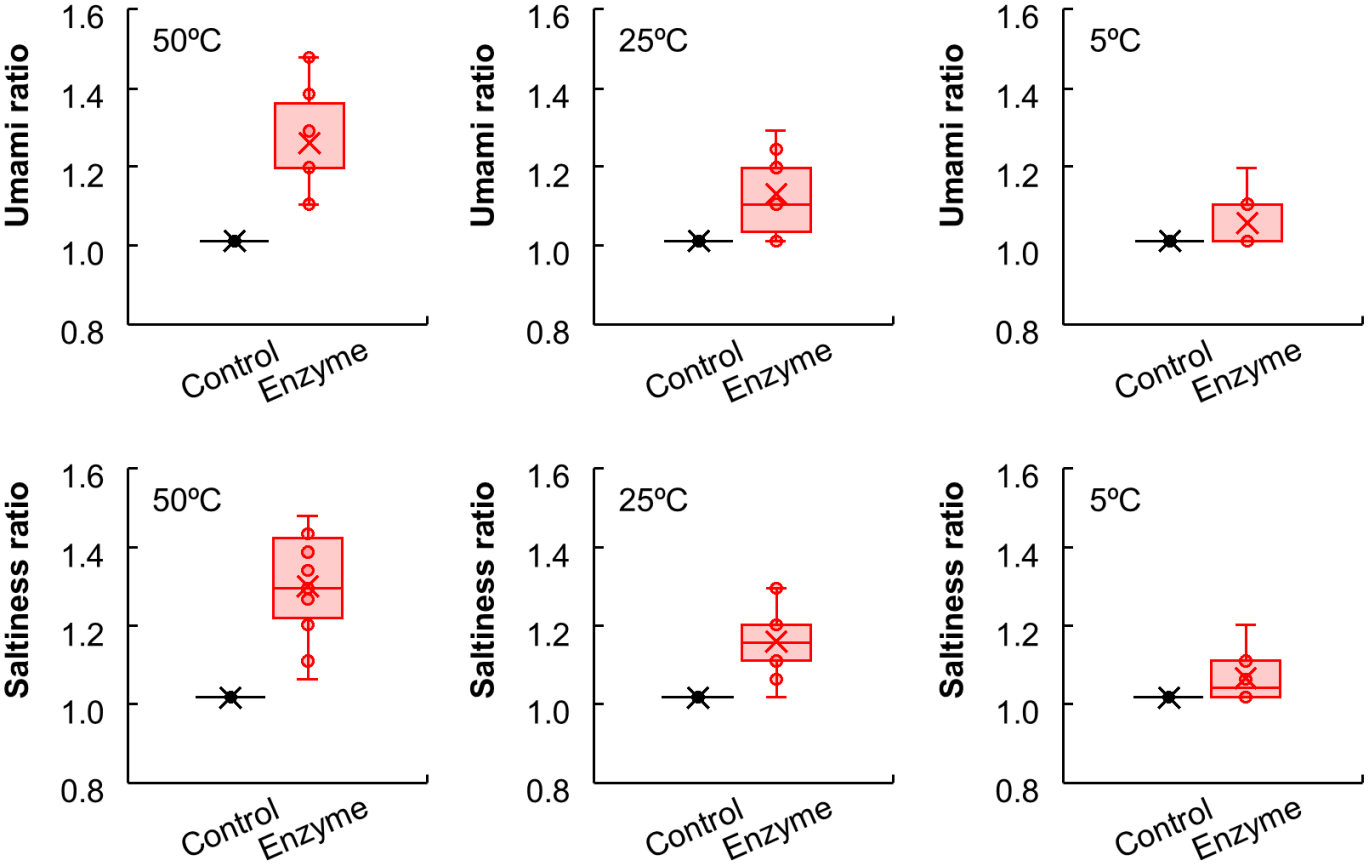
The effects of temperature on umami and saltiness of vegetable soup. Sensory evaluations conducted on control and enzyme-treated vegetable soups at 50°C, 25°C, and 5°C. Umami and saltiness scores of enzyme-treated vegetable soups compared to control soup.

Furthermore, we investigated the salt reduction rate of the enzyme-treated vegetable soup compared with the control soup following NaCl supplementation (Figure 2C). The saltiness intensity ratio of the control soup supplemented with 0.50% NaCl was set to 1.0, and compared with this, the ratio of the enzyme-treated soup supplemented with 0.50, 0.45, 0.40, and 0.35% NaCl were 1.31, 1.16, 0.99, and 0.79, respectively in the sensory evaluation. Compared with the control soup with 0.50% NaCl, the saltiness intensity of the enzyme-treated soup with 0.50 and 0.45% NaCl was significantly higher (*p* < 0.01); however, the saltiness intensity of the enzyme-treated soup with 0.40% NaCl was comparable to that of the control soup. As expected, regardless of enzyme addition, NaCl content in vegetable soup remained unchanged (Figure 2D). Interestingly, the effects of the enzyme blend on saltiness and umami enhancement were more pronounced when the temperature of the vegetable soup was increased (Figure 3). This finding suggests that enzymatic treatment with protease and glutaminase may be an effective tool for achieving a 20% salt reduction rate.

The rate of salt reduction by saltiness-enhancing flavorants is an important indicator for the practical development of salt-reduced food products. Some previous studies have reported salt reduction rates equal to or greater than 20% (Figure 2C) using enzymes: 60% reduction using Glu (1), 30% reduction using octapeptides derived from *Agaricus bisporus* (51), 25% reduction using proteases derived from *Lentinula edodes* (52), 24% reduction using disodium succinate (14), and 25–30% reduction using pyroglutamyl tripeptides and organic acids (53). While adding these enhancers to actual final foods can be effective strategy, the feasibility may be constrained by factors such as their concentration levels, manufacturing and purification costs, taste, and regulatory considerations as food additives. Notably, such strategies are unable to meet the recent clean-label demands to reduce the amount and number of food additives, and an increasing number of consumers tend to avoid food products containing additives (21). However, the enzyme blend and their reaction products in this study are not considered as additives because the enzymes were deactivated during the processing of the vegetable soup. Therefore, enzyme blends of proteases and glutaminases could be a novel tactic to enhance the saltiness levels of final food products while meeting clean-label requirements.

### 3.4 Identifying the saltiness-enhancing tastants in enzyme-treated vegetable soup with added sodium chloride

**Figure 4:**
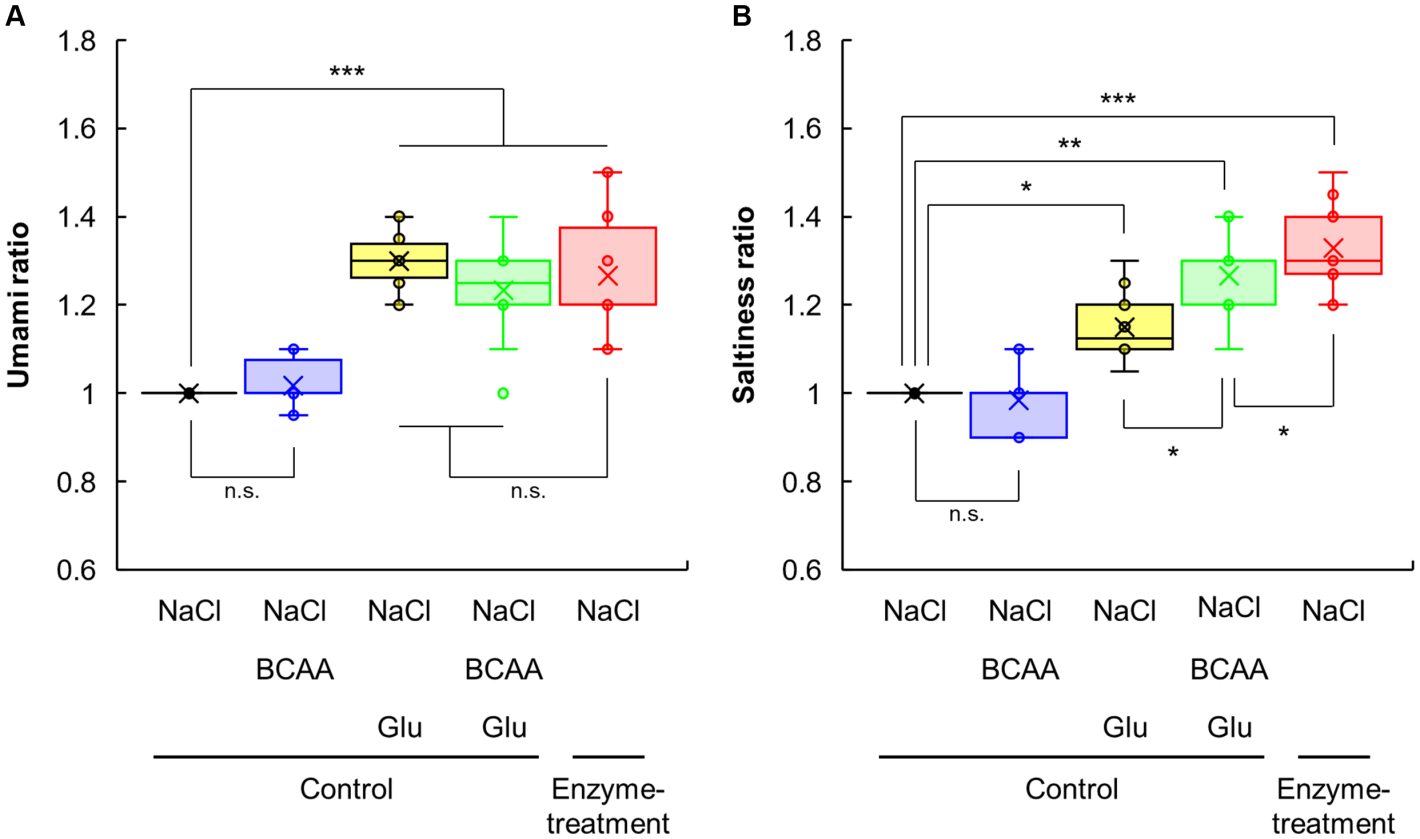
The effects of amino acid addition on umami and saltiness of vegetable soup. Sensory evaluations of enzyme-treated vegetable soup in the presence of NaCl, Glu, and/or BCAA, compared with the umami **(A)** and saltiness **(B)** of the control soup.

Next, we explored the saltiness enhancement tastants in enzyme-treated vegetable soup (Figure 4; Supplementary Figure S2). As shown in Figures 1C,D, the protease and glutaminase blend produced amino acids and peptides. Because fractionation and collection of peptides in the soup were difficult, we evaluated the effect of taste–taste interactions by amino acids (His, Ser, Val, Ala, Leu, Gly, Asp, Ile, and Glu) that were significantly increased by enzyme treatment (Figure 1C). Glu addition significantly enhanced the umami intensity of the control soup after 0.5% NaCl supplementation, and this intensity was comparable to that of the enzyme-treated soup containing 0.5% NaCl (Figure 4A). Although Glu addition significantly enhanced the saltiness of the control soup containing 0.5% NaCl, this intensity was lower than that of the enzyme-treated soup containing 0.5% NaCl (Figure 4B). Interestingly, after adding all nine amino acids increased by the enzyme treatment to the control soup containing NaCl, the saltiness levels were significantly increased compared with the control soup containing NaCl and Glu (Supplementary Figure S2A). These findings suggest that amino acids other than Glu molecules that can enhance saltiness exist in enzyme-treated vegetable soups.

The interaction of amino acids expressing the five basic tastes with taste receptors generally depends on the structure (size, charge, and polarity) of their side chain (45). Thus, amino acids increased by enzyme treatment were classified according to their chemical and taste properties, and we searched for other amino acids that have a saltiness enhancement effect of NaCl through taste–taste interactions, except for Glu. Accordingly, nine amino acids were classified into five groups: (i) polar basic amino acid (bitter), His; (ii) polar acidic amino acid (umami), Asp and Glu; (iii) polar neutral amino acid (sweet), Ser; (iv) non-polar amino acid (sweet), Ala and Gly; and (v) nonpolar amino acid (bitter), Val, Leu, and Ile (Supplementary Figure S2B). Considering various combinations of these groups, soup containing a combination of BCAAs (Val, Leu, and Ile) and Glu had a higher saltiness intensity of NaCl (Supplementary Figure S2A; Figure 4). BCAA addition did not affect the umami taste of the control soup containing 0.5% NaCl irrespective of the presence of Glu (Figure 4A). Similarly, BCAA addition did not affect the saltiness of the control soup containing 0.5% NaCl. However, compared with Glu or BCAA addition alone, adding both BCAA and Glu significantly enhanced the saltiness of control soup with 0.5% NaCl (Figure 4B). These findings indicate that the combination of BCAA and Glu could be an important factor for enhancing the saltiness intensity of NaCl.

Saltiness is perceived by several receptors and ion channels, especially epithelial sodium channels, transient receptor potential vanillic acid, and transmembrane channel-like 4 (30, 51, 52). However, our knowledge regarding the receptor structures and interactions between saltiness-enhancing flavorants and receptors is limited, and hence, there is a lack of research on saltiness-enhancing flavorants at the molecular level (30). Some previous studies, using an electronic tongue evaluation method, have reported that certain amino acids, such as Glu or BCAA, can enhance saltiness (10, 54). Recently, a study using molecular docking simulation and electronic tongue evaluation reported that Glu molecules under neutral conditions have a negative charge and can bind to the transmembrane channel-like 4 receptor, thereby enhancing saltiness intensity (55). Another recent study using molecular docking simulation and sensory evaluation has reported that Glu and constituent amino acid residues are important for binding to the transmembrane channel-like 4 receptor and enhancing salty taste in saltiness-enhancing peptides (DIQPEER, DEPLIVW, and LPDEPSR) identified from mushrooms (52). Based on these findings, as well as the present study’s results, we suggest that Glu and BCAAs released by enzyme blends can interact with saltiness receptors, increasing the saltiness intensity of NaCl in vegetable soups. In contrast, the saltiness intensities of control soups containing 0.5% NaCl supplemented with all nine amino acids or Glu + BCAA were similar but significantly lower than those of the enzyme-treated soup containing 0.5% NaCl. Thus, components other than BCAA and Glu that enhance the salty taste may exist in enzyme-treated vegetable soups. However, further research is required to identify saltiness-enhancing peptides through taste–taste interactions because the peptides produced by enzyme blends are difficult to isolate (Figure 1D).

### 3.5 Sensory evaluation of enzyme-treated vegetable soup with added potassium chloride

**Figure 5:**
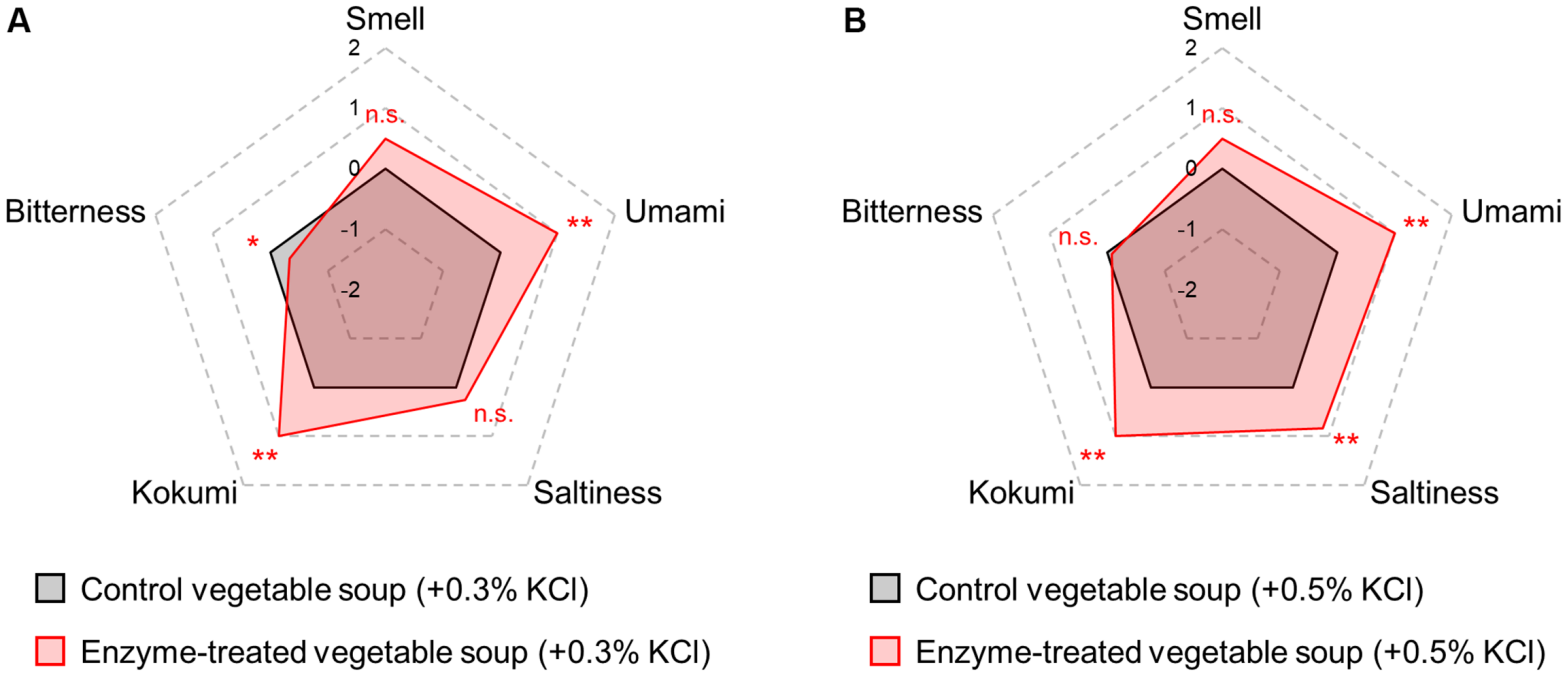
Sensory profiles of vegetable soup added with KCl. Sensory evaluations of control and enzyme-treated vegetable soups conducted in the presence of 0.3% **(A)** and 0.5% **(B)** KCl final concentration.

Another approach for reducing the salt content is the addition of salt replacers other than saltiness-enhancing flavorants. Among salt replacers, potassium chloride (KCl) is the most popular choice as a feasible salt substitute (56). In particular, salt reduction obtained by the proportional replacement of NaCl with KCl is the easiest method for manufacturers. However, consumer acceptance is limited owing to its pronounced bitter or metallic taste and aftertaste (57). Generally, the umami taste of Glu is able to mask the bitterness intensity in food (38). Thus, we investigated the ability of the Glu molecules released by the blend of proteases and glutaminase to reduce KCl bitterness intensity (Figure 5). Compared with each control soup, 0.3% KCl addition to the enzyme-treated soup slightly enhanced the saltiness intensity (*p* > 0.05), and 0.5% KCl addition significantly enhanced (*p* < 0.01). Compared with each control soup, enzyme treatment significantly reduced the bitterness intensity caused by 0.3% KCl (*p* < 0.05) but did not affect the bitterness intensity of 0.5% KCl. These findings indicate that treatment with enzyme blends can suppress the characteristic bitterness of KCl, when its concentration is approximately ≤0.3%.

Many studies have reported that Glu molecules decrease bitterness levels (29, 38, 58–60). However, some studies also have reported results that contradict these results and this is potentially caused by the differences in bitter substances, their concentrations, and test methods in the sensory test (61, 62). Of them, the intensities and combinations of Glu and bitter substances might be important factors. One recent study reported that Glu molecules did not enhance or suppress other tastes when other tastes were displayed at relatively stronger intensities (62). Generally, the thresholds for KCl bitterness are 0.06% in an aqueous solution and 0.10% in an emulsion state similar to that found in food (63), and bitterness intensity of KCl tends to further increase depending on the concentration (64). As shown in Figure 5, the bitterness of 0.3% KCl relatively close to the bitter threshold was suppressed by Glu presence but the bitterness of 0.5% KCl bitterness was not. Therefore, it is suggested that the ability of Glu to mask KCl bitterness might be affected by the KCl concentration (bitterness intensity). Although there are few reports about the masking effects of Glu on the bitter tastes of food containing NaCl partially replaced by KCl, one previous study reported that bitterness induced by 0.15–0.35% KCl could be suppressed by Glu addition (65). Nevertheless, there is currently limited research and knowledge on the range of KCl bitterness intensity (or its concentration) that the Glu molecule can decrease.

## 4 Conclusion

In this study, we optimized the enzymatic reaction conditions for a mixture of bacterial and fungal proteases and glutaminase and then investigated the synergistic effects of this enzyme blend on the umami and saltiness levels of vegetable soup. The activities of proteases and glutaminase in the vegetable soup were not detected after deactivation. Sensory evaluations indicated that the reaction products (e.g., protein hydrolysates or amino acids) enhanced the umami, kokumi, and saltiness of vegetable soups containing 0.5% NaCl. Notably, the saltiness intensity ratio of the enzyme-treated soup with 0.50, 0.45, and 0.40% NaCl was 1.31-, 1.16-and 0.99-fold higher compared with that of control soup, achieving a 20% salt reduction rate. Moreover, the enzyme-produced Glu and BCAA synergistically enhanced the saltiness intensity of NaCl. These findings suggest that enzyme blends of bacterial and fungal proteases and glutaminase can be used to enhance the saltiness of vegetable soups while meeting clean-label requirements.

## Data Availability

The original contributions presented in the study are included in the article/Supplementary material, further inquiries can be directed to the corresponding author.
