# Supplementary material for: Umami and saltiness enhancements of vegetable soup by enzyme-produced glutamic acid and branched-chain amino acids
Source: Front Nutr. 2024 Aug 19;11:1436113. doi: 10.3389/fnut.2024.1436113 (PMC11368061; doi:10.3389/fnut.2024.1436113)
Supplement: Supplementary file 1 [file Image_1.pdf]

Supplementary Material

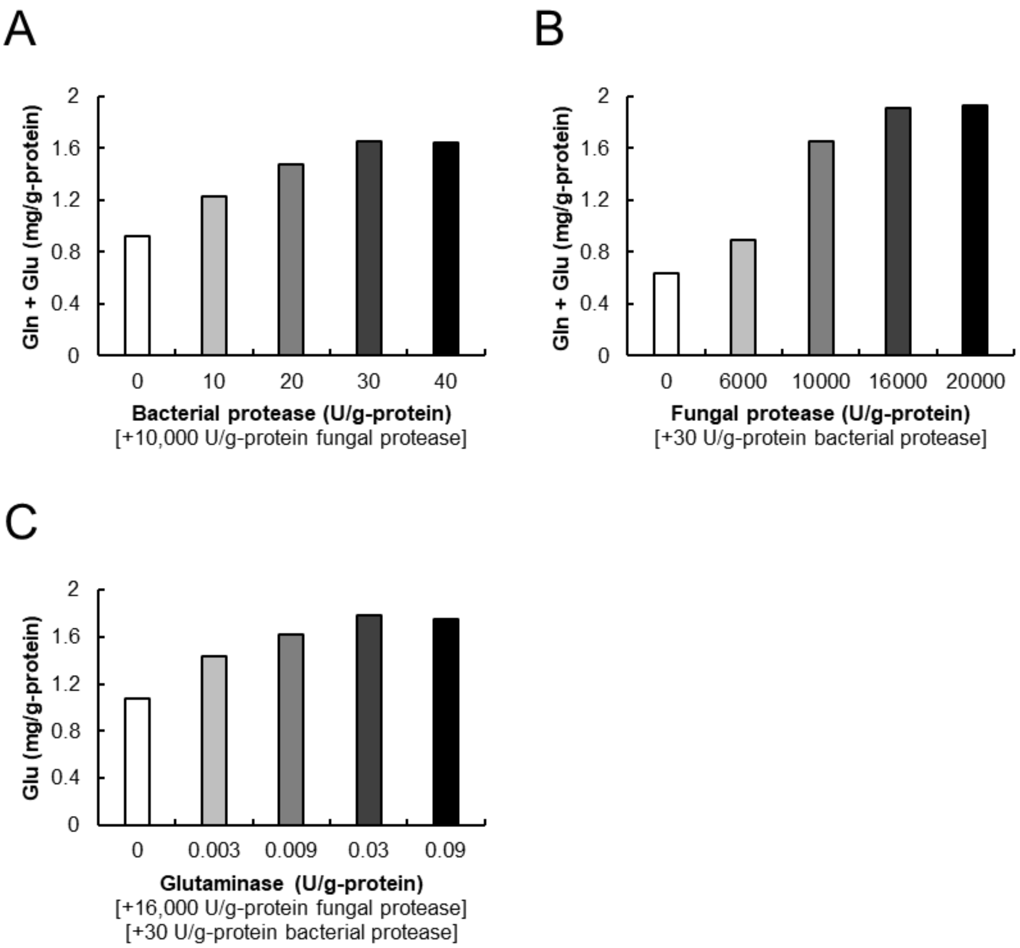

Supplementary Figure 1. Optimization of enzyme ratio for glutamic acid production. (A) Optimization of bacterial protease amounts for producing Gln and Glu in the presence of fungal proteases. (B) Optimization of fungal protease amounts for producing Gln and Glu in the presence of bacterial proteases. (C) Optimization of glutaminase amounts producing Glu in the presence of bacterial and fungal proteases.

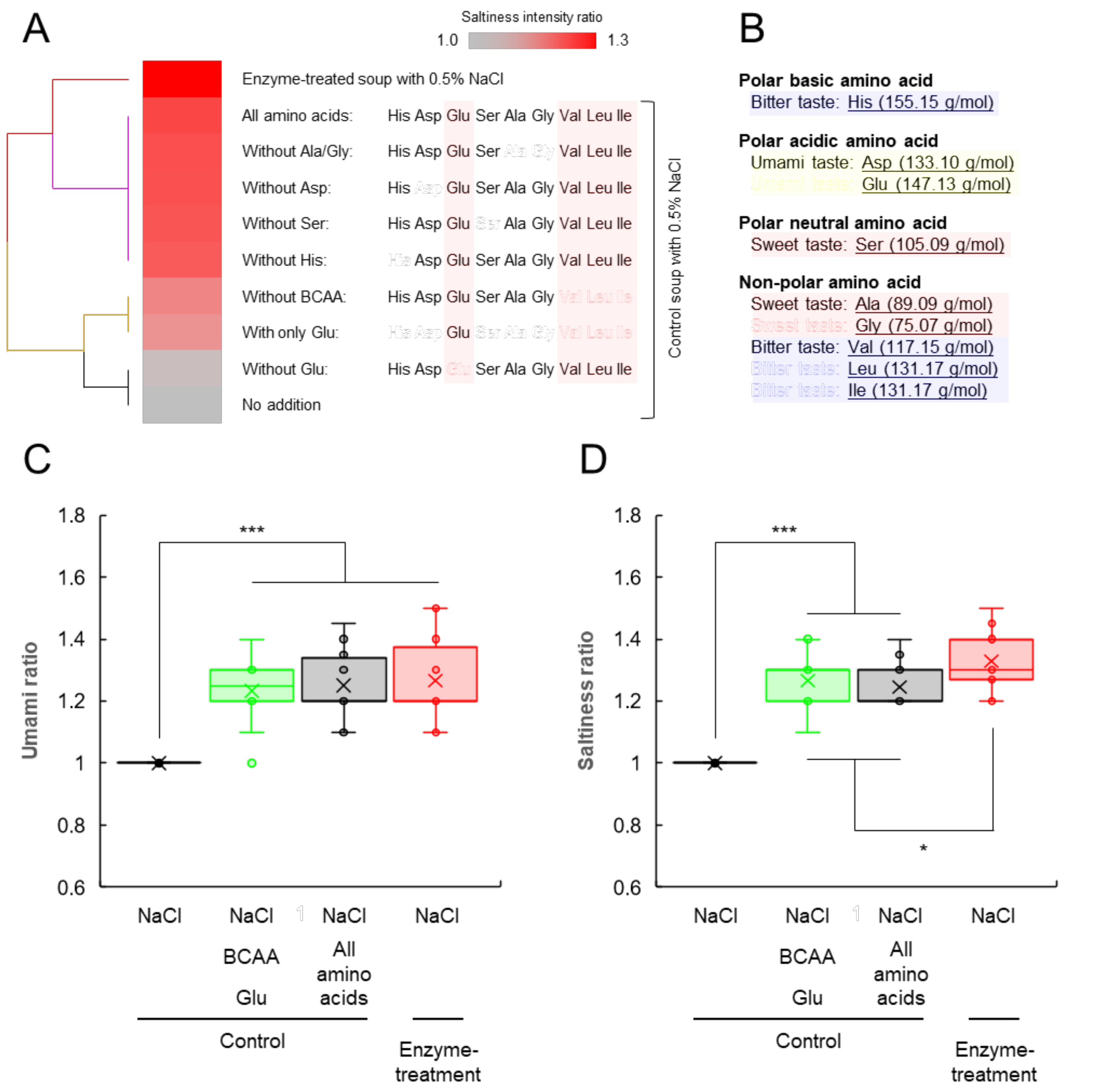

Supplementary Figure 2. Exploration of saltiness-enhancing amino acids. The saltiness intensity ratio of the vegetable soup containing various combinations of amino acids was scored through sensory evaluations.
